# Dichroism of coupled multipolar plasmonic modes in twisted triskelion stacks

**DOI:** 10.1515/nanoph-2025-0063

**Published:** 2025-07-21

**Authors:** Javier Rodríguez-Álvarez, Joan Vila-Comamala, Antonio García-Martín, Albert Guerrero, Xavier Borrisé, Francesc Pérez-Murano, Christian David, Álvaro Blanco, Carlos Pecharromán, Xavier Batlle, Arantxa Fraile Rodríguez, Amílcar Labarta

**Affiliations:** Departament de Física de la Matèria Condensada, 16724 Universitat de Barcelona, 08028 Barcelona, Spain; Institut de Nanociència i Nanotecnologia (IN2UB), 16724 Universitat de Barcelona, Barcelona, 08028, Spain; Paul Scherrer Institute, Forschungsstrasse 111, Villigen 5232, Switzerland; Instituto de Micro y Nanotecnología IMN-CNM, CSIC, CEI UAM + CSIC, Isaac Newton 8, 28760, Tres Cantos, Madrid, Spain; Institut de Microelectrónica de Barcelona (IMB-CNM, CSIC), Bellaterra, 08193, Spain; Catalan Institute of Nanoscience and Nanotechnology (ICN2), CSIC and BIST, Campus UAB, Bellaterra, 08193 Barcelona, Spain; Instituto de Ciencia de Materiales de Madrid (ICMM), Consejo Superior de Investigaciones Científicas (CSIC), Calle Sor Juana Inés de la Cruz 3, Madrid, E-28049, Spain

**Keywords:** plasmonics, chirality, dichroism, triskelion, twisted stack

## Abstract

We present a systematic investigation of the optical response to circularly polarized illumination in twisted stacked plasmonic nanostructures. The system consists in two identical, parallel gold triskelia, centrally aligned and rotated at a certain angle relative to each other. Sample fabrication was accomplished through a novel multilevel high-resolution electron beam lithography. This stack holds two plasmonic modes of multipolar character in the near-infrared range, showing a strong dependence of their excitation intensities on the handedness of the circularly polarized incident light. This translates into a large circular dichroism which can be modulated by adjusting the twist angle of the stack. Fourier-transform infrared (FTIR) spectroscopy and numerical simulations were employed to characterize the spectral features of the modes. Remarkably, in contrast to previous results in other stacked nanostructures, the system’s response exhibits a behavior analogous to that of two interacting dipoles only at small angles. As the angle approaches 15°, where maximum dichroism is observed, more complex modes of the stack emerge. These modes evolve towards two in-phase multipolar excitations of the two triskelia as the angle increases up to 60°. Finally, simulations for a triangular array of such stacked elements show a sharp mode arising from the hybridization of a surface lattice resonance with the low-energy mode of the stack. This hybridized mode demonstrates the capability to be selectively switched on and off through the light polarization handedness.

## 1 Introduction

Chirality is an intrinsic geometrical property of objects that cannot be superimposed with their mirror image. This property gives rise to two distinct realizations, termed enantiomers, which exhibit opposite handedness. Although often overlooked, chirality plays a crucial role in numerous critical phenomena, particularly in the field of biochemistry. The handedness of biological molecules such as amino acids, DNA or enzymes, for instance, has been demonstrated to be of paramount importance in elucidating their properties [1], [2]. A remarkable example is limonene, where one enantiomer is responsible for the characteristic fragrance of citrus fruits, while its counterpart contributes significantly to the scent of numerous coniferous and broadleaved trees [3].

Chirality is not limited to physical objects but can also be a property of fields, as is the case with left-handed (LCP) and right-handed (RCP) circularly polarized light [4], [5]. Light with a specific handedness serves as a valuable probe for chiral media, since light–matter interaction can be dictated by the handedness of both entities [6], [7], when present. Such chiroptical activity is commonly quantified through the optical functions *f* of the system under the two circular polarizations by means of the circular dichroism (CD) that can be defined as:

(1) $$\text{C}{\text{D}}_{f}=\frac{f_{\text{LCP}}-f_{\text{RCP}}}{f_{\text{LCP}}+f_{\text{RCP}}},$$

where *f* stands for any of the optical cross-sections (CS), namely, the absorption, scattering, and extinction, under either RCP or LCP illumination.

The chiral interaction in naturally occurring systems is typically of low magnitude, presenting substantial challenges for detection and quantification. Consequently, engineered chiral plasmonic structures have gained relevance because of their enhanced light–matter interaction associated with large values of the near fields. They show enhancements of the chiral signal of several orders of magnitude while not being affected by the inherent lossy behavior of plasmonic resonances [8], reaching an enhancement factor of 150 for Ag dimers [9] and up to 800 using Au nanorods [10], among others [11], [12], [13], [14]. Furthermore, their integration into hybrid nanostructures promises active control of chirality [15].

Nowadays, strategies to fabricate chiral nanostructures are very diverse. However, due to the inherent difficulty to manufacture 3D structures using conventional nanofabrication techniques, early approaches were based on planar plasmonic structures [16], [17], [18], [19], [20]. These planar configurations typically exhibited limited chiroptical activity due to their two-dimensional nature, as truly planar structures cannot manifest chiroptical activity without violating system reciprocity [21]. Consequently, the observed chiroptical activity in many such structures was primarily attributed to their interaction with the substrate, effectively breaking the planar approximation [17], [19], [22]. Alternative designs have relied on the interaction between two modes through the excitation of Fano resonances [23], [24]. While these systems present circular dichroism (CD) in the absorption and the scattering CS, the opposite sign of those contributions result in mutual cancellation. As a result, the extinction CS remains polarization-independent, again in accordance with reciprocity principles [25].

Further developments in nanofabrication technology have enabled the realization of diverse 3D nanostructures showing large values of CD, such as spirals [26], helicoidal arrangements [27], [28], and various stacked planar nanostructures ranging from nanorods [29], [30], crosses [31], split ring resonators [32], [33], and gammadions [32], [34], [35], among others. Over the last decade, twisted plasmonics has emerged as a distinct field of research, driven by the large chiroptical activity and tunability achievable through stacked nanostructures [36] or van der Waals materials [37].

In this work, we present a twisted stack formed by two identical planar nanostructures, called triskelia, which are characterized by their inherent 2D chirality and threefold rotational symmetry. In contrast to previously reported systems [27], [28], [29], [31], [32], [33], the monomers within our stack exhibit intrinsic chiroptical activity. Furthermore, their threefold symmetry hinders the excitation of simple modes with an even number of poles while promoting the mixing of higher-order multipolar modes. As elucidated in our previous work [38], the small distance between the stack elements facilitates strong inter-element interactions, resulting in the emergence of handedness-dependent resonances in the near infrared spectral region. This configuration gives rise to pronounced CD which can be modulated by adjusting the twist angle between the stacked elements. Here, we investigate the nature of those resonances as a function of the twist angle between the two stacked structures, demonstrating that higher-order multipolar modes play a crucial role in the excitation of the structure under circularly polarized illumination. Our study is based on Fourier transform infrared spectroscopy (FTIR) measurements and finite difference time domain (FDTD) simulations.

## 2 Triskelion stack

**Figure 1:**
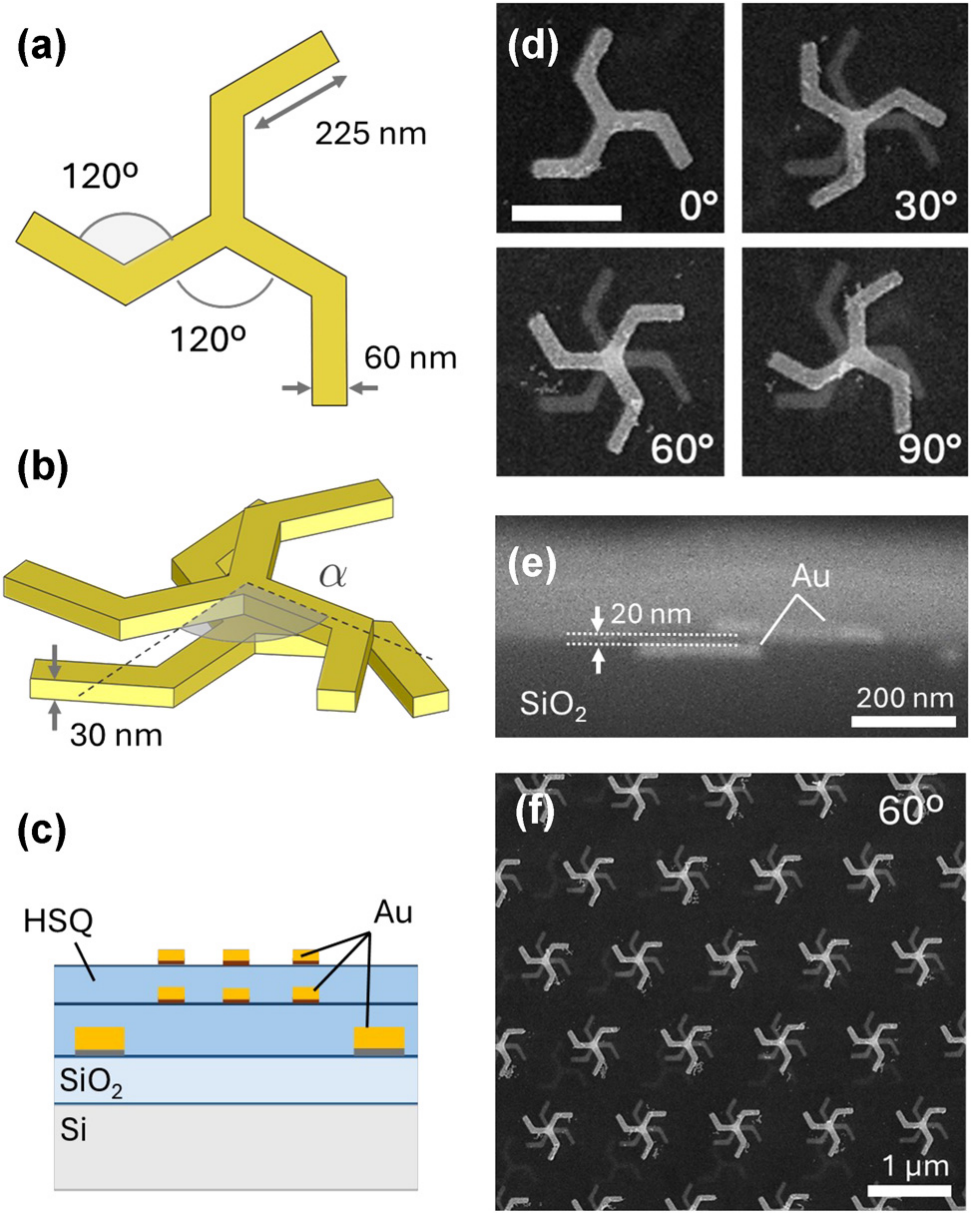
Schematic depiction of a triskelion displaying the dimensions of the studied nanostructure (a), a stack indicating the twist angle *α*, and the layers that form the structure, including the substrate and alignment markers (c). Scanning Electron Microscopy (SEM) images showing a top view of selected fabricated nanostructures (d), a cross-section image of a representative sample (e), and the spatial arrangement of one of the fabricated triangular arrays of stacked triskelia with a pitch of 1,200  nm, designed to explore the hybridization of the stack modes with a surface lattice resonance (SLR) (f). Scale bar is 400 nm.

The motif constituting the building block of the stack, the triskelion, is designed as three identical gold elements extending from a common central point, with each element oriented at 120° intervals. These elements exhibit a clockwise bend of 120° at their respective midpoints, thereby preserving threefold rotational symmetry while eliminating mirror planes parallel to the axis of rotation (see Figure 1a). This geometric arrangement confers 2D chirality to the triskelion structure, as shown in [38]. The specific dimensions and geometry of the studied monomer are shown in Figure 1a. The thickness of the planar structure was set to 30 nm. The final 3D structure consisted of a twisted stack of two coupled plasmonic triskelia, a schematic of which is shown in Figure 1b, where the twist angle *α* is defined so that the stack has right-handed chirality whenever *α* < 60°. Note that the chirality of the stack reverses (becoming left-handed) for *α* > 60° due to the monomer’s threefold symmetry. The arm bending breaks the symmetry between clockwise and anticlockwise twisting directions, rendering symmetrical angle values around 60° inequivalent, unlike structures with straight arms, where such symmetry is preserved. *α* = 60° is a symmetrical configuration without a distinct handedness. Samples with various twist angles were manufactured through multiple exposures of electron beam lithography (EBL), all of them with a vertical separation between the two stacked elements of about 20 nm. The tunability of the stack’s twist angle is static and it is achieved during fabrication via three lithography steps. The first step involves patterning alignment markers, which are critical as a reference for subsequent layers of triskelia. The second lithography step creates the first triskelion layer, using the alignment markers as a reference. The third lithography step produces the second layer of triskelia, completing the twisted stacks. Precise alignment between the centers of triskelia in both layers must be maintained using the same markers and parameters as in the first step. Given the inherent complexity of this multistep lithography process, optimal twist angles were pre-determined through numerical simulations. The stacks were arranged in a triangular array with variable pitch. Additional information regarding the manufacturing process is shown in the Methods section. Some examples of the manufactured stacks are shown in Figure 1d. The cross-sectional image in Figure 1e indicates good parallelism and consistent separation between the two triskelia. To prevent the symmetry breaking caused by substrate interactions, the stack was embedded in a homogeneous medium with a refractive index of *n* = 1.42, ensuring uniform dielectric interfaces. Figure 1f shows a magnified view of a representative sample with a small pitch (1,200 nm), designed to explore the hybridization of the stack modes with a surface lattice resonance (SLR), and demonstrates the microstructural uniformity of the fabricated stacks.

## 3 Results and discussion

**Figure 2:**
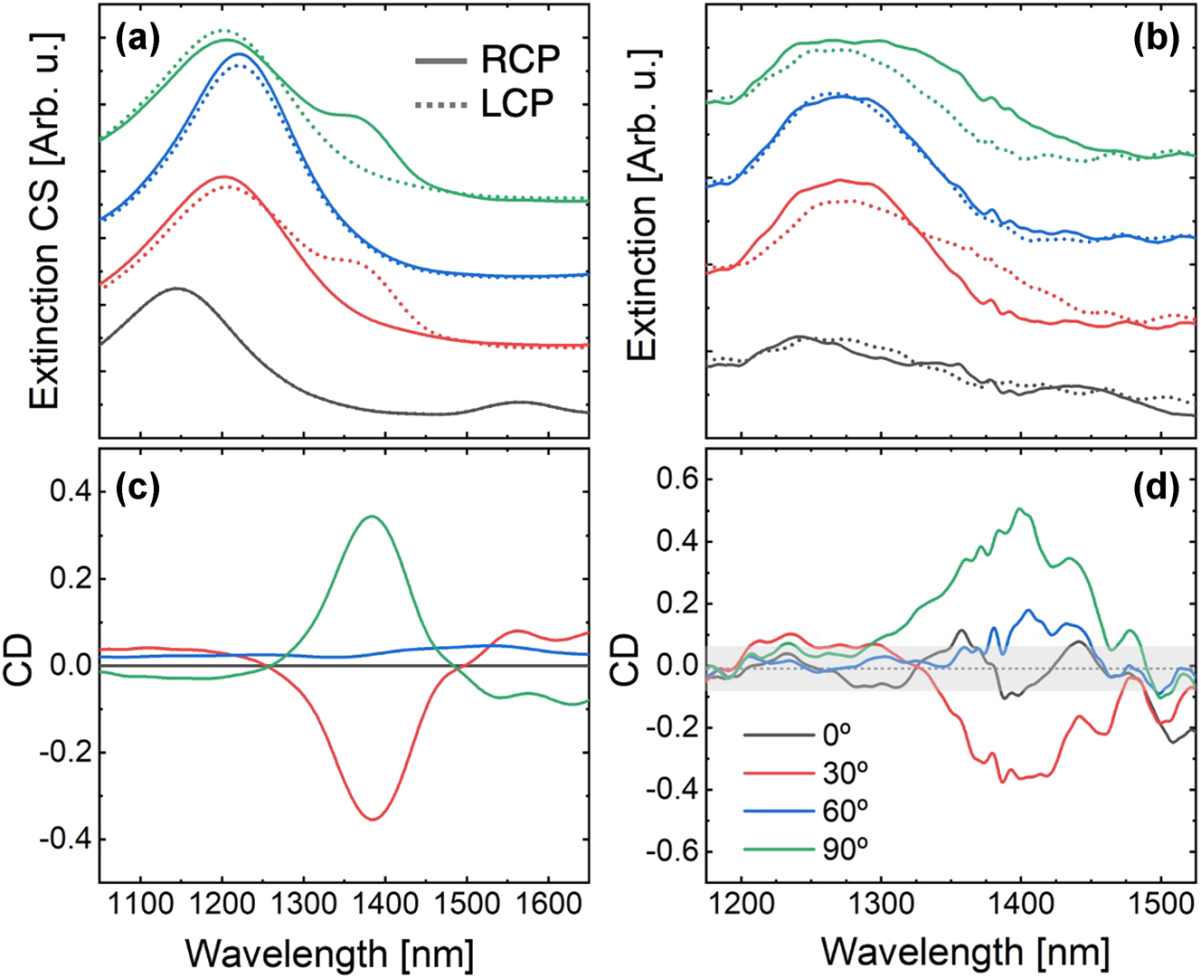
Simulated (a) and measured (b) extinction spectra for 0°, 30°, 60°, and 90° (black, red, blue, and green, respectively), under LCP (solid line) and RCP (dashed line) illumination. The baselines of the spectra are shifted arbitrarily for clarity. Simulated (c) and experimental (d) CD extracted from the data in panels (a) and (b), respectively, using Eq. (1). The shaded area in panel (d) indicates the typical size of the error bar for the CD, calculated as the root mean square deviation from 0 for the CD at 0°.

The structure exhibits two plasmonic resonances in the wavelength range between 1,100 and 1,600 nm, resulting from the hybridization of a multipole resonance in each triskelion. Both modes show strong signals in the absorption cross-section, whereas the scattering cross-section for the lower-energy mode is severely suppressed, as shown in Figure S1 in the Supplementary Information. Thus, both FTIR measurements and simulations reveal two extinction peaks within this range, which undergo remarkable spectral shifts and intensity variations as the twist angle is modified. As illustrated in Figure 2a and b, the intensity of these peaks, particularly the one at lower energy, is strongly influenced by the handedness of the circularly polarized incident radiation. As a result, these handedness-dependent resonances yield large CD, as shown in Figure 2c and d. The calculated near-field distributions for 30° and 90° are presented in Figure S2 in the Supplementary Information as representative examples. It is worth noting that the excitations studied are of multipolar character and not as intense as the primary dipolar excitations (far in the infrared range, approximately 3,700 nm, for this structure, see Figure S3 in the Supplementary Information). Despite this, there is a general qualitative agreement between the experimental and simulated curves in Figure 2, including the opposite sign of the CD observed for the 30° and 90° cases.

Moreover, owing to the threefold symmetry of the triskelion, both the spectral shifts and intensity variations are similar around 60° when the handedness of the light polarization is exchanged. However, due to the arm bending, CD is slightly higher at 90° than at 30°. At 90°, both the arm bending and twist direction synergistically enhance the stack’s chirality, whereas at 30°, these contributions partially cancel each other. Despite having a small effect in the extinction CS, the differences in the optical response for both angles are much prominent when the absorption and scattering CS are considered separately. In particular, at 90°, CD in the extinction for the low energy mode originates mainly from CD in the absorption CS due to a large reduction of the scattering signal, unlike for the 30° case (see Figure S4 in the Supplementary Information). Furthermore, arm bending introduces an additional asymmetry relative to the monomer’s threefold axis, hampering the excitation of simple modes with an even number of poles symmetrically distributed around the center of the system. As a result, the associated near-field distribution becomes more dispersed within the stack volume (see color maps in Figure S2 in the Supplementary Information), leading to an overall reduction in the net dipole moment at energies around and below the out-of-phase mode, and therefore, a depression in the scattering CS. The effect of the arm bending is further illustrated in Figure S5 in the Supplementary Information, which compares the optical response of a triskelion stack with that of a stack based on a three-straight-arm monomer, both with identical volume and a twist angle of 30°. Notably, this reduction in the scattering CS of the triskelion stack enhances its CD signal within a narrower wavelength range, thus improving its practical applicability.

**Figure 3:**
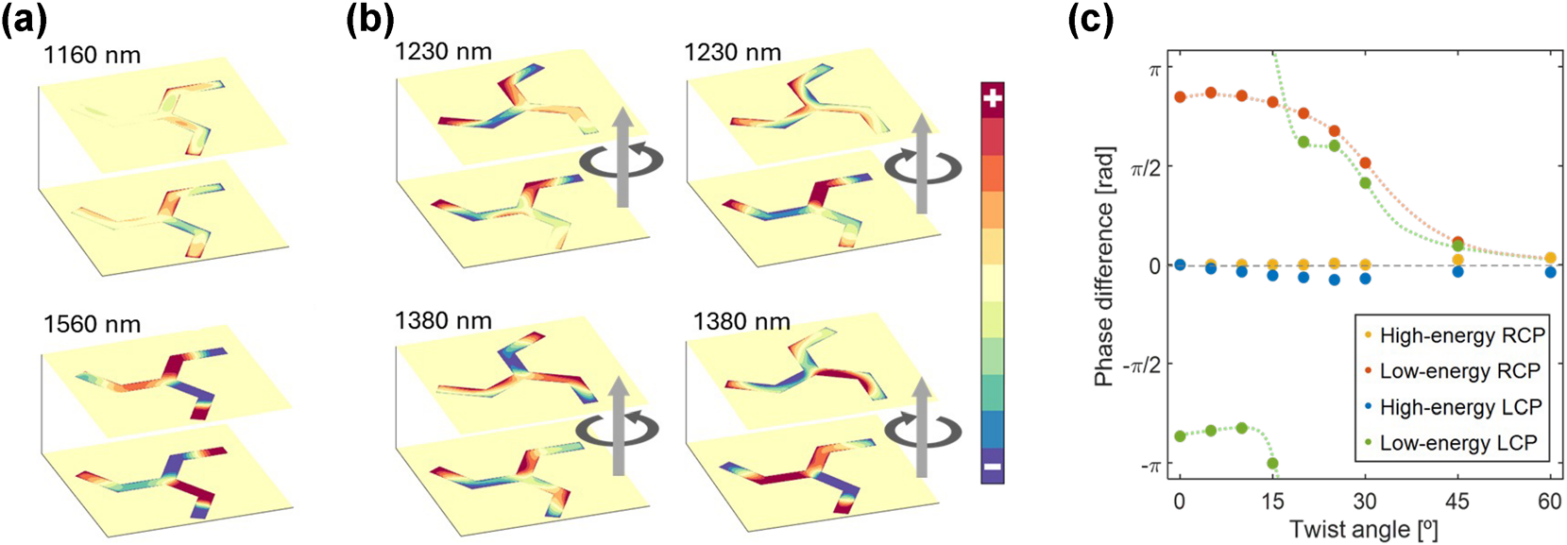
Simulated charge distributions for facing triskelion interfaces for twist angles of 0° (a) and 30° (b). The simulations at different angles correspond to arbitrary values of the phase of the incoming illumination. Panel (c) presents the phase difference between the electric dipole moment orientations of the top and bottom triskelia for each twist angle. All the information displayed shares a common scale. For the 0° case, only a single polarization is presented, as the response is identical for both LCP and RCP. The geometry of the illumination, indicated by vertical gray arrows, corresponds to that of the FTIR measurements.

The typical approach to describing the optical response of a chiral medium would be to consider a simple and straightforward framework: the Born–Kuhn model [39], [40], [41]. This approximation supposes two interacting electrons oscillating in orthogonal directions in parallel planes separated a distance *d*. The coupling between the two particles results in two new eigenstates of the coupled oscillators system. This model yields results analogous to those of the hybridization of two plasmonic modes [42], [43] and has been experimentally validated for plasmonic systems [44]. However, in prior studies, *d* was of the order of the excitation wavelength, while in the present work, *d* ∼ 20 nm. As a result, the phase shift of the incident radiation between the two monomers is rather small, weakening the chiral response. Simultaneously, since the interaction between plasmonic elements is mediated by their near fields, the close proximity between the two monomers in our study significantly enhances their coupling. This strategy has been exploited by many works and is one of the main benefits of stacked systems with spacings significantly smaller than the exciting wavelength [15], [29], [30], [31]. Given these considerations, a model based solely on the phase shift, such as the Born-Kuhn model, cannot fully account for the optical response of the triskelion stack. We propose instead an interpretation based on the hybridization of the plasmonic modes of individual triskelia. As shown in the snapshots of the charge distributions of the triskelia in Figure 3, the complexity of the motif gives rise to multipolar excitations, especially fostered by geometric frustration; specifically, the threefold symmetry of the triskelion does not effectively accommodate an even number of poles, which are typically associated with simple dipole or multipolar modes. These plasmonic resonances, in turn, interact with those of the second triskelion in the stack resulting in many hybrid modes that deviate from the ideal in-phase/anti-phase excitations often considered in previous works involving two interacting dipoles [19], [33]. Moreover, the geometry of the system changes with the twist angle, favoring the excitation of low-energy resonances displaying variable dephasing between the polarizations of the two triskelia. This is clear in Figures 3c and S6 in the Supplementary Information, where charge distributions and computed dipole moments for the two components of the stack as a function of *α* for the high- and low-energy modes are shown. Thus, while the high-energy mode (1,230 nm) maintains the two electric dipole moments relatively in-phase as *α* changes, the low energy mode (1,380 nm) exhibits a variable dephasing between the polarizations of both triskelia in the stack. Only at very small angles, the low-energy mode is sufficiently close to the anti-phase polar configuration (see Figures 3a, c and S6 in the Supplementary Information). Around 30°, the polarizations of the two triskelia become perpendicular in the low-energy mode (see Figures 3b, c and S6 in the Supplementary Information). As the angle *α* further increases, the polarizations corresponding to the low- and high-energy modes begin to resemble each other more closely (see Figures 3c and S6 in the Supplementary Information). This observation indicates that, although only two peaks are detected for any value of *α*, the features of the low-energy mode excited in each case are significantly different.

These findings suggest that the chiroptical activity in the stack arises from the dependence of the excitation intensity of these modes on the light-handedness, in accordance with previously reported results [29], [31], [38]. However, in this case, the resonances yielding CD are not sufficiently explained by the simplistic model involving the splitting of a triskelion mode into anti-phase and in-phase modes of the stack. Instead, they are better characterized by the excitation of an out-of-phase mode that is highly sensitive to the geometry of the stack at each angle *α*, in conjunction with the in-phase excitation. This continuous shift of the dephasing between the dipole moments of the monomers in the stack is shown in Figures 3 and S6 in the Supplementary Information, for angles between 0° and 60°. In addition, the small net dipole moments of the out-of-phase, low-energy resonances agree with the fact that CD is typically associated with the selective excitation of modes exhibiting poor scattering [38].

**Figure 4:**
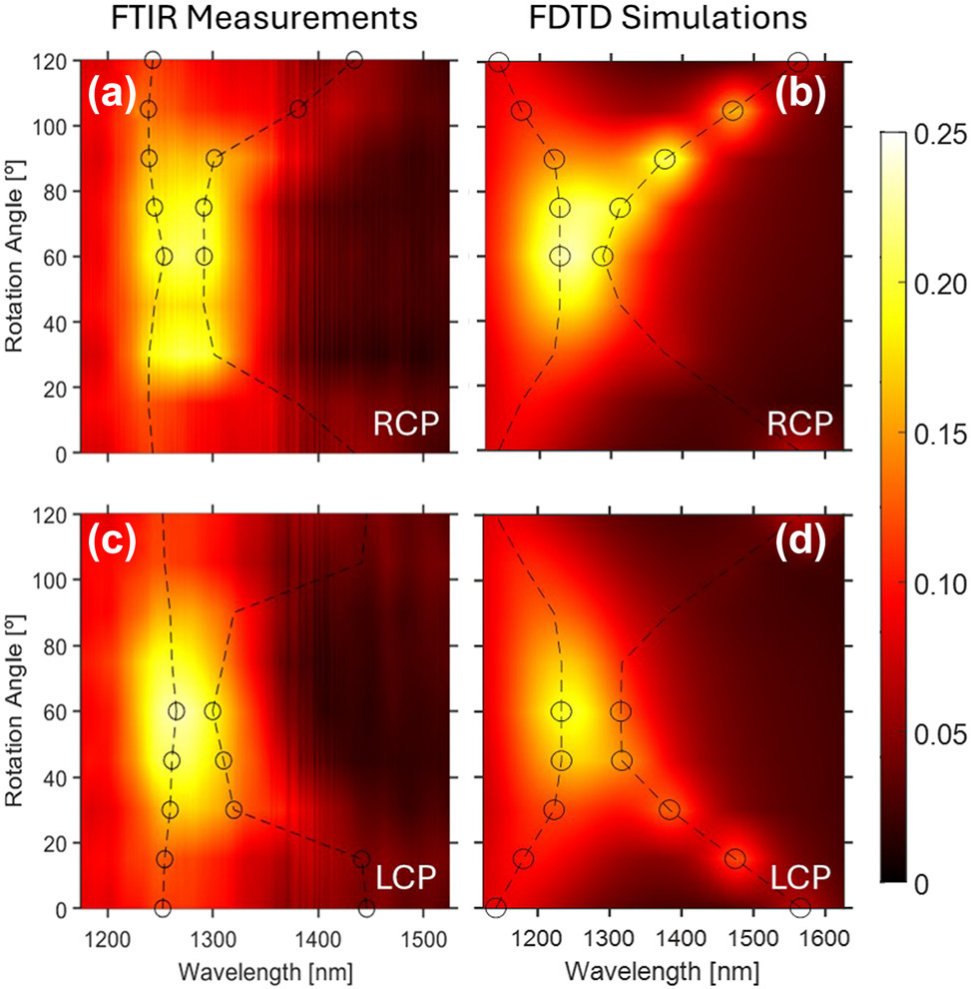
Colormaps depicting the experimental (a, c) and simulated (b, d) extinction spectra for twist angles between 0° and 120° for both RCP (a, b) and LCP (c, d). Empty circles highlight the spectral positions of the two Lorentzian peaks fitted to both the experimental and simulated data. Dashed lines serve as guidelines to the eye.

In Figure 4, the spectral response of the stack as a function of *α* is shown in colormaps obtained after linear interpolation from FTIR and FDTD data for twist angle values that are integer multiples of 15°. Remarkably qualitative agreement between both sets of colormaps is found. Two Lorentzian functions, corresponding to the low- and high-energy resonances, were fitted to both the experimental and simulated spectra, with the position of some of these peaks also displayed for comparison in Figure 4. The details of the fitting are presented in Figure S7 in the Supplementary Information.

For angles between 0° and 60°, the structure favors the excitation of the out-of-phase, low-energy mode for the light-handedness opposite to that of the stack, i.e. LCP since the chirality of the stack is right-handed. Consequently, the out-of-phase mode is most efficiently excited by circular polarization that induces the greatest change in the relative orientation of the electric field with respect to the lower and upper triskelia (note that illumination is from bottom to top). In contrast, RCP light will excite both triskelia similarly due to the minimal phase shift of the light across *d*, thereby favoring the in-phase mode and significantly hindering the out-of-phase resonance. At 60°, the excitation features of the system are quite similar under both circular polarizations of the light, with only slight variations arising from the rightward bending of the triskelion branches. Despite this minor splitting at this angle, the intensity of the low-energy resonance is nearly negligible, as shown in Figure S7 in the Supplementary Information, resulting in a single prominent peak in the extinction spectra. For angles greater than 60°, symmetric optical responses are observed with respect to those for *α* < 60° under reversed circular polarizations, except for small differences in the intensity of the low-energy mode. This slight asymmetry is owed to the fact that the triskelion is a chiral object in 2D, thus slightly altering the expected symmetric response around 60° due to its inherent threefold symmetry.

**Figure 5:**
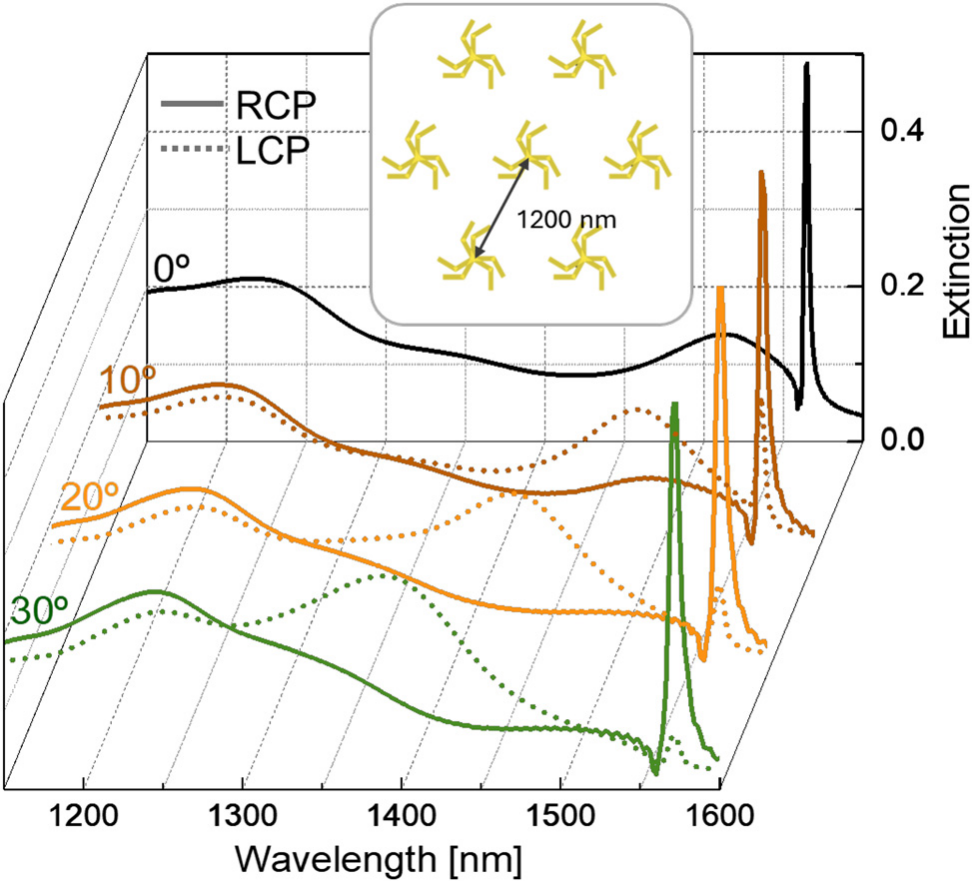
Simulated extinction spectra for an array of stacked triskelia with a pitch of 1,200 nm, embedded in a homogeneous medium of *n* = 1.5 with a separation of 50 nm between triskelia. The response of the array under RCP and LCP illumination is presented for different values of the twist angle. The inset shows a schematic depiction of the simulated array of twisted stacks of triskelia nanostructures.

Finally, the excitation of a SLR by circularly polarized light in a triangular array of these stacked structures was investigated by numerical simulation (see Figure 5 inset). The array pitch was set to 1,200 nm to position the SLR at a wavelength slightly redshifted relative to the out-of-phase peak of the stack at 0° (where splitting of the two coupled modes is maximized). Interestingly, we found that the intensity of this SLR strongly depended on the handedness of the circular polarization of the light and the twist angle, resulting in very large dichroism for angles larger than 10°. These results were obtained from numerical simulation of an array embedded in a medium of *n* = 1.5, where each element consisted of a twisted stack with 50 nm in separation between the two monomers and a twist angle range of 0° ≤ *α* ≤ 30°. Figure 5 shows the simulated extinction CS under RCP and LCP light at four selected values of *α*, with a fixed pitch of 1,200 nm. For this pitch and refractive index, a SLR emerges as a sharp peak with a Fano profile located around 1,550 nm, precisely in the region where anti-phase excitations of the triskelion stack become energetically favorable (as seen in the case for *α* = 0° in Figure 5). Therefore, the Fano resonance originates from the hybridization of the SLR mode with the anti-phase plasmonic excitation of the stack, where the sharp and intense peak of the resonance corresponds to the coherent excitation of the out-of-phase mode across the array of triskelion stacks through absorption of the incoming radiation. Consequently, the cooperative interaction among the near fields created by the elements in the array, due to the geometrical resonance condition, amplifies the excitation of the anti-phase mode in every stack (which would otherwise be very weak), leading to a large energy absorption. This excitation occurs with much greater efficiency when illuminated by light that matches the handedness of the stack (LCP in this case), as incoming radiation with the handedness of the stack favors a larger polarization of the first triskelion. This, in turn, induces a larger anti-phase polarization in the second triskelion through near-field interactions. It is important to note that in-phase modes cannot be excited in this range of relatively low energies. Under circular polarization with opposite handedness to that of the stack (RCP in this case), polarizations induced in both triskelia within each stack are reduced, leading to lower absorption associated with the lattice resonance. As a result, the spectra in Figure 5 exhibit large differences in the intensity of the SLR modes under RCP and LCP illumination, resulting in high values of dichroism, except at *α* = 0° where the stack lacks chirality. The SLR is robust to slight changes of the refractive index. Nevertheless, different refractive index values will yield a different resonant wavelength.

We attempted to experimentally confirm these results involving the coupling of the SLR and the anti-phase plasmonic resonance of the stack, using samples like those shown in Figure 1f. However, we did not find such a sharp peak, but a family of low intensity broad peaks in the spectral region of interest. Finite-size broadening effects may arise from several factors including inadequate perpendicular illumination of the samples due to the numerical apertures of the optical systems used for light focusing, insufficient translational symmetry in the array, and variations in the patterned features across fabricated samples [45].

## 4 Conclusions

We have presented a comprehensive study of the chiroptical response of a twisted stack of triskelion nanostructures, emphasizing the detailed characterization of two coupled multipolar modes in the near-infrared region. Our findings, supported by FTIR measurements and FDTD simulations, demonstrate that both the spectral position and intensity of the resonances can be precisely tuned by adjusting the angle between the two elements in the stack. The three-fold symmetry of the elements promotes complex multipolar excitations which are incompatible with an even number of pairs of equal poles with opposite sign. In particular, under illumination with an opposite handedness to that of the structure, two multipolar resonances are observed; conversely, illumination with light that matches the handedness of the structure reveals only the peak corresponding to the high-energy excitation. This results in large dichroism in the extinction CS of the system, except at the particularly symmetric twist angles of 0° and 60°.

Our findings show that the high-energy mode originates from an in-phase excitation of the two triskelia under both RCP and LCP illumination. In contrast, the low-energy mode, which is enhanced by illumination under opposite handedness to that of the stack, corresponds to the excitation of the two monomers with a specific phase difference between their instantaneous polarizations. Thus, contrary to previous works, we demonstrate that anti-phase oscillations occur only at small twist angles; for angles greater than 15°, the polarizations of the two monomers progressively align in phase as *α* increases towards 60°. This draws a significantly more complex scenario depending on the twist angle, diverging from the descriptions provided by the Born–Kuhn model or the hybridization of two equivalent plasmonic resonances producing simple in phase- and antiphase-coupled modes of the stack.

Finally, simulations indicate that arranging such a chiral stack in a plasmonic triangular lattice of the appropriate pitch leads to the selective excitation of a SLR depending on the light handedness. This configuration enables high CD values within a narrow wavelength range, that can be tuned by the pitch of the lattice and the geometry of the stack.

## 5 Methods

### 5.1 Sample fabrication

Sample manufacturing was performed at the Laboratory for X-ray Nanoscience and Technologies at the Paul Scherrer Institut (Switzerland). Electron beam lithography (EBL) is the technique of choice for producing 2D planar structures with high resolution and features sizes down to a few nanometers and can also achieve 3D structuring of more complex structures through multiple exposures [46]. The samples were fabricated by three consecutive EBL steps, following the sequence depicted in Figure S8 in the Supplementary Information. The samples were lithographed on a Si substrate (250 µm thick) coated with a SiO_2_ layer on both sides (1.8 µm thick, deposited by wet thermal oxidation). The initial EBL step was aimed at defining the alignment markers, which consisted of Au squares of 100 × 100 nm^2^ and 80 nm in thickness using an adhesion layer of 10 nm of Cr. The marker pattern was exposed in PMMA resist, followed by an electron beam evaporation of Cr and Au, and was completed by a lift-off process. The thickness of these markers has proven to be critical for their correct detection, and therefore, alignment of the EBL system for further lithography processes. The second step was to cover the markers with hydrogen silsesquioxane (HSQ) resist in solution in methyl isobutyl ketone (MIBK), commercially labelled as FOx (flowable oxide) [47], [48]. This diluted polymer was deposited through spin coating, pre-baked at 80 °C for 4 min and then annealed at 400 °C for a minimum of 1 h. This produced a homogeneous layer of SiO_2_ of 300 nm in thickness approximately following a cost-effective and controllable protocol.

Subsequently, an additional EBL step was carried out to pattern the first layer of triskelia, using the alignment markers as a reference. Following the exposure of the triskelia pattern in PMMA resist and development, 30 nm of Au was deposited by electron beam evaporation on top of a 2 nm adhesion layer of Ge, which has been shown to better preserve the plasmonic properties of noble metal nanostructures compared to other common materials, such as Cr or Ti [49]. After completing the lift-off process, another layer of HSQ was deposited and then annealed, resulting in a layer with a thickness of approximately 50 nm.

Finally, the third and final EBL step was performed. A second layer of twisted triskelia was patterned, ensuring that the centers of the triskelia in both layers were aligned, using the same alignment markers and parameters as in the previous step. Following this complex process, the two layers of twisted triskelia, separated by 20 nm, were embedded in SiO_2_. Interestingly, despite the substrate consisting of a relatively thick layer of Si, the transparency of the entire double-oxidized Si wafer is approximately 50 %, particularly for wavelengths larger than 1,150 nm (see Figure S9 in the Supplementary Information).

EBL was performed using a Raith/Vistec EBPG 5000Plus with a Gaussian-shaped beam, offering a maximum writing field size of 1,024 × 1,024 μm^2^ and high beam stepping frequencies of up to 125 MHz, and operating at 100 keV acceleration and with an overlay precision around 20 nm for larger write fields and 10 nm for smaller ones (100 × 100 μm^2^). The metallization was performed using physical vapor deposition (PVD) by evaporating the target using an electron gun.

### 5.2 FDTD simulations

The FDTD simulations presented in this work were performed using the commercial software provided by Lumerical [50]. All simulations were performed using a 2 × 2 × 2 nm^3^ cell size setting a homogeneous background refractive index of 1.42 or 1.5 without absorption to better reproduce the optical properties of annealed HSQ. The refractive index and absorption coefficient for Au were obtained from ref. [51]. The circularly polarized illumination was introduced using linearly polarized plane waves by setting two sources with orthogonal polarization angles and a *π*/2 phase difference between them. Two types of simulations were performed, those featuring single stacked elements, and others where the response of an infinite triangular lattice of stacked elements was simulated.

The former relies on the use of total-field scattered-field (TFSF) sources and perfect absorbing boundary conditions. Absorption and scattering CS data were collected since only one element was being simulated. For these simulations the extinction CS was the sum of absorption and scattering CS. The second type of simulations requires the use of plane wave sources and periodic boundary conditions at the in-plane boundaries. In this case, reflection and transmission through the structure were measured. The extinction in this framework was assigned to be 1 − *T*, where *T* stands for the transmission CS. It is worth noting that the reflection of the structure was very small compared to the extinction signal.

### 5.3 FTIR measurements

A Bruker Vertex 70V Spectrophotometer with a Hyperion 2000 microscope was used to measure the extinction spectra using RCP and LCP polarizers. Transmission through the sample was measured using 4× magnification to minimize the numerical aperture of the system and maximize normal incidence.

## Supplementary Material

Supplementary Material Details
