# Supplementary material for: Dichroism of coupled multipolar plasmonic modes in twisted triskelion stacks
Source: Nanophotonics. 2025 Jul 21;14(17):2847–56. doi: 10.1515/nanoph-2025-0063 (PMC12397745; doi:10.1515/nanoph-2025-0063)
Supplement: Supplementary file 1 — Supplementary Material Details [file j_nanoph-2025-0063_suppl_001.pdf]

Supplementary Information for:

Dichroism of coupled multipolar plasmonic modes in twisted triskelion stacks

Javier Rodríguez-Álvarez[1,2,], Joan Vila-Comamala[3], Antonio García-Martín[4], Albert Guerrero[5], Xavier Borrisé[6], Francesc Pérez-Murano[5], Christian David[3], Álvaro Blanco[7], Carlos Pecharromán[7], Xavier Batlle[1,2], Arantxa Fraile Rodríguez[1,2], and Amílcar Labarta[1,2].*

[1] Departament de Física de la Matèria Condensada, Universitat de Barcelona, 08028 Barcelona, Spain

[2] Institut de Nanociència i Nanotecnologia (IN2UB), Universitat de Barcelona, 08028, Spain

[3] Paul Scherrer Institute, Forschungsstrasse 111, Villigen 5232, Switzerland

[4] Instituto de Micro y Nanotecnología IMN-CNM, CSIC, CEI UAM + CSIC, Isaac Newton 8, 28760, Tres Cantos, Madrid, Spain

[5] Institut de Microelectrónica de Barcelona (IMB-CNM, CSIC), Bellaterra, 08193, Spain

[6] Catalan Institute of Nanoscience and Nanotechnology (ICN2), CSIC and BIST, Campus UAB, Bellaterra, 08193 Barcelona, Spain

[7] Instituto de Ciencia de Materiales de Madrid (ICMM), Consejo Superior de Investigaciones Científicas (CSIC), Calle Sor Juana Inés de la Cruz 3, Madrid, E-28049 Spain

*E-mail: javier.rodriguez@ub.edu;

- **S1.** Simulated absorption and scattering cross-section for a single triskelion and a stack of triskelia for 0º of twist angle.
- **S2.** Simulated near field for 30º and 90º configurations
- **S3.** Extended spectral response of a single triskelion and charge distribution for the dipolar mode.
- **S4.** Simulated absorption and scattering cross-section for a stack of triskelia for 90º and 30º of twist angle.
- **S5.** Comparison between the optical response of a triskelion stack and that based on a monomer with three straight arms.
- **S6.** Electric dipole moment for both triskelia for the high- and low-energy resonances for several twist angles.
- **S7.** Lorentzian fitting of the measured and spectra for several twist angles.
- **S8.** EBL lithography steps involved in the fabrication of the structures.
- **S9.** Calculated transmission of the substrate.

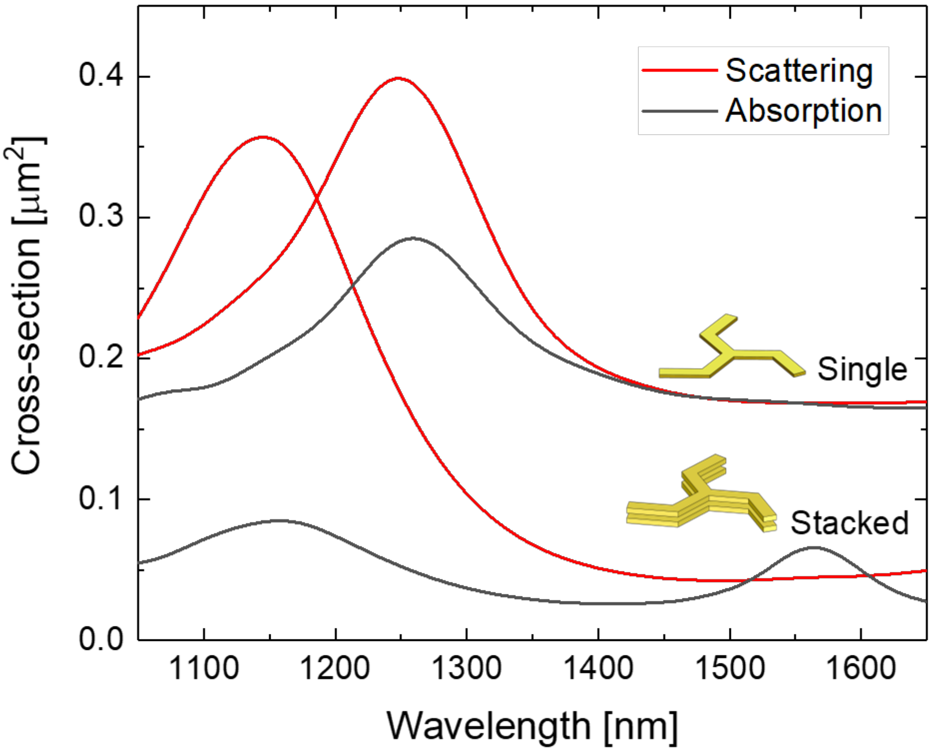

Fig. S1: Spectra of the simulated absorption and scattering cross-sections for a single triskelion and for a stack of two triskelia with a twist angle of 0° and a spacing of 20 nm between them. The interaction between the two elements in the stack results in a splitting of the original resonance, producing two resonances at higher and lower energies. Note that the low-energy resonance exhibits an almost negligible scattering signal. Note that the spectra for the single triskelion are shifted vertically for clarity.

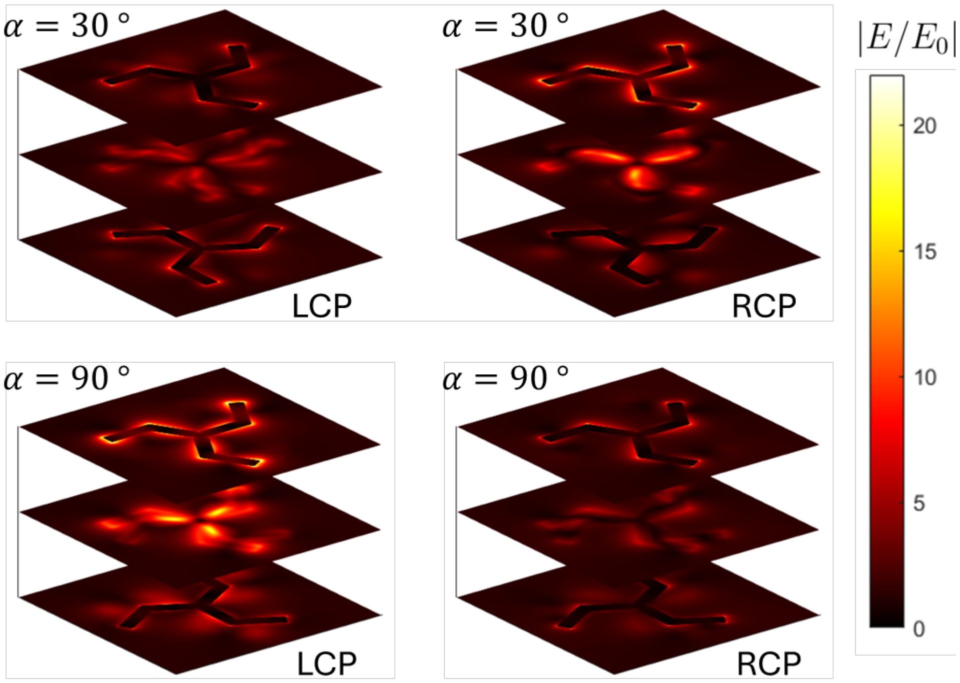

Fig. S2: Colormaps for simulated near fields at the low-energy resonance (around 1380 nm) for two values of the twist angle, under LCP and RCP illumination. Near fields are represented in two parallel planes intersecting both elements in the stack and in a third plane in the middle between them. The electric field is normalized to the incident field.

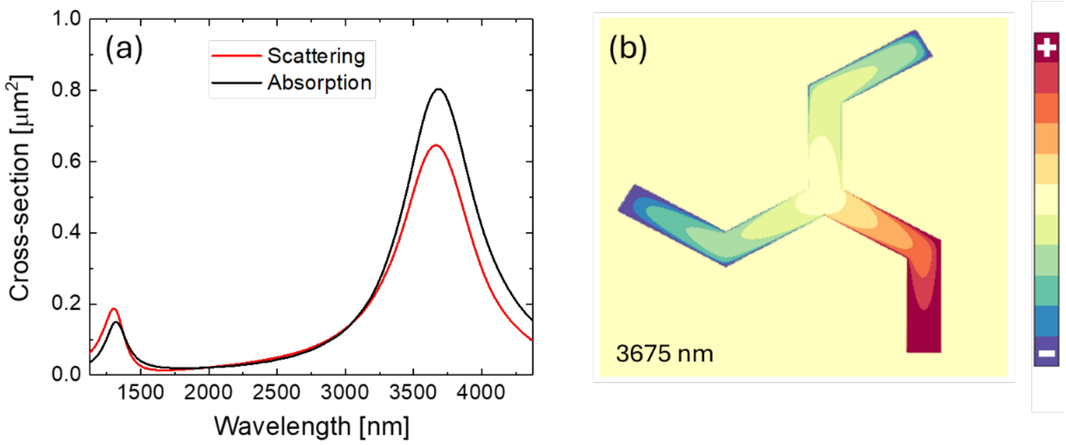

Fig. S3: (a) Simulated absorption and scattering cross-sections for a single triskelion over an extended wavelength range. A dominant resonance is observed at 3675 nm, with significantly higher intensity than the multipole excitation peak around 1250 nm. (b) Real part of the charge density (at an arbitrary phase) for the 3675 nm resonance, revealing its predominantly dipolar character. Note that the threefold symmetry of the triskelion induces geometric frustration in the charge density distribution, even for this simple dipole mode.

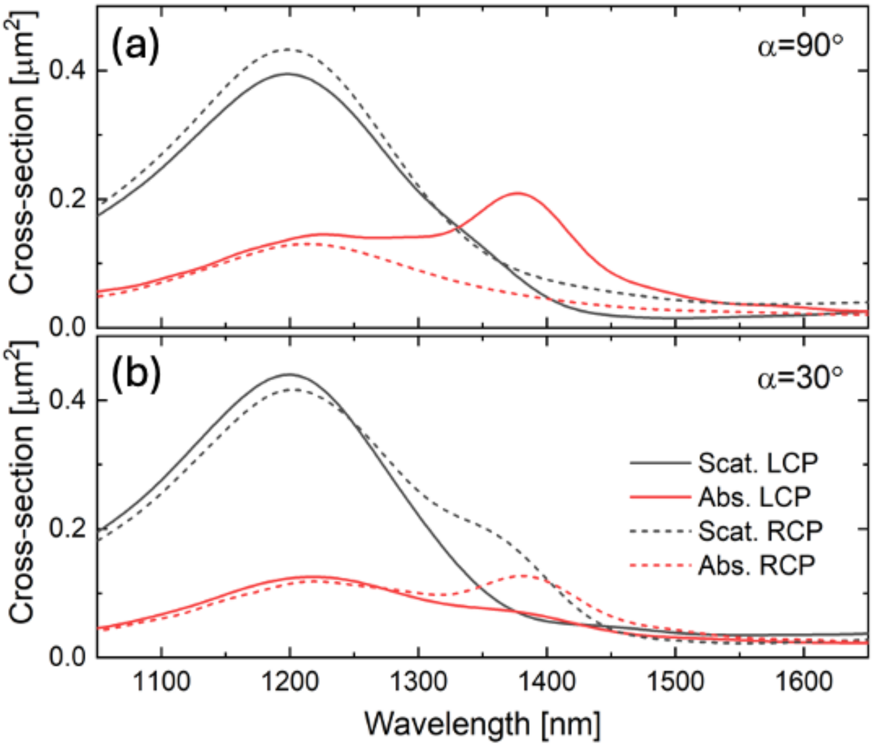

Fig. S4: Simulated scattering and absorption cross-sections under LCP and RCP illumination for a triskelion stack with a twist angle of (a) 90° and (b) 30°.

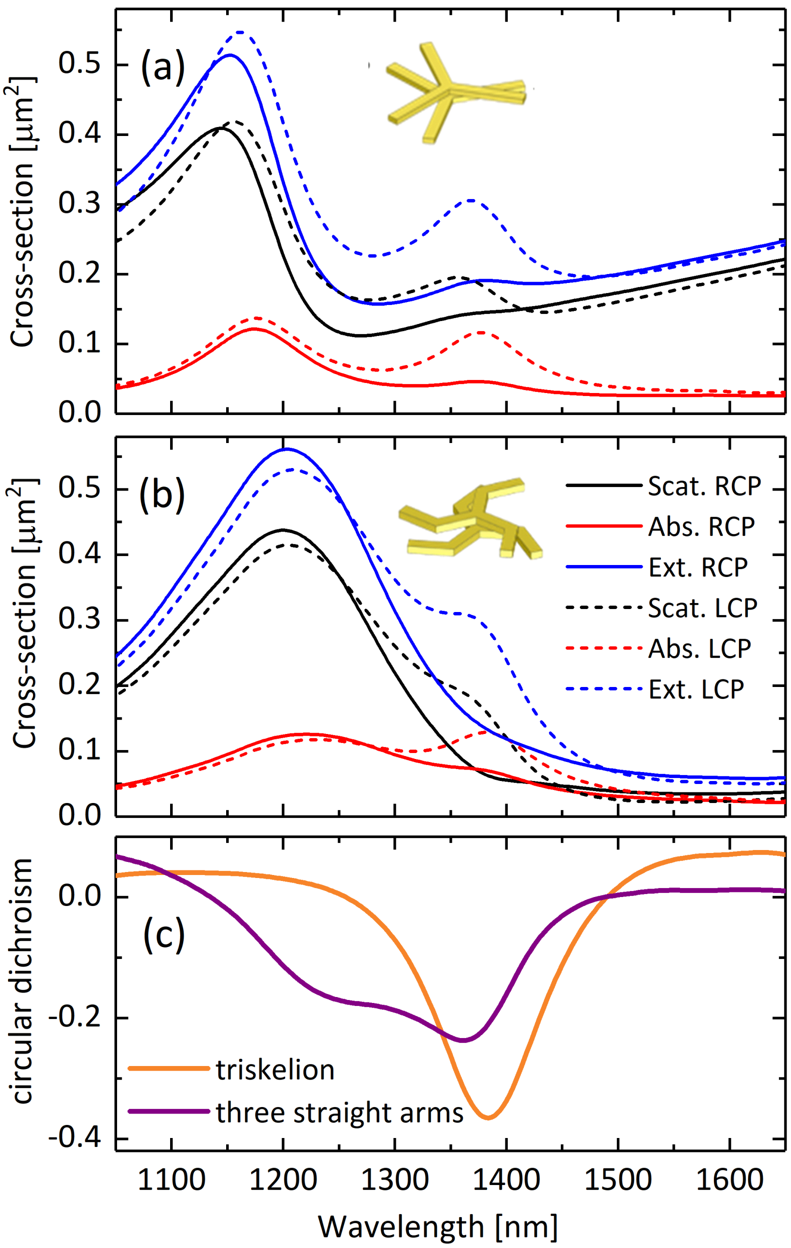

Fig. S5: Simulated scattering, absorption, and extinction cross-sections under LCP and RCP illumination for a triskelion stack (a) and one formed by a monomer with three straight arms (b), both with a twist angle of 30° and equal volumes. (c) Corresponding CD in the extinction cross-section of panels (a) and (b).

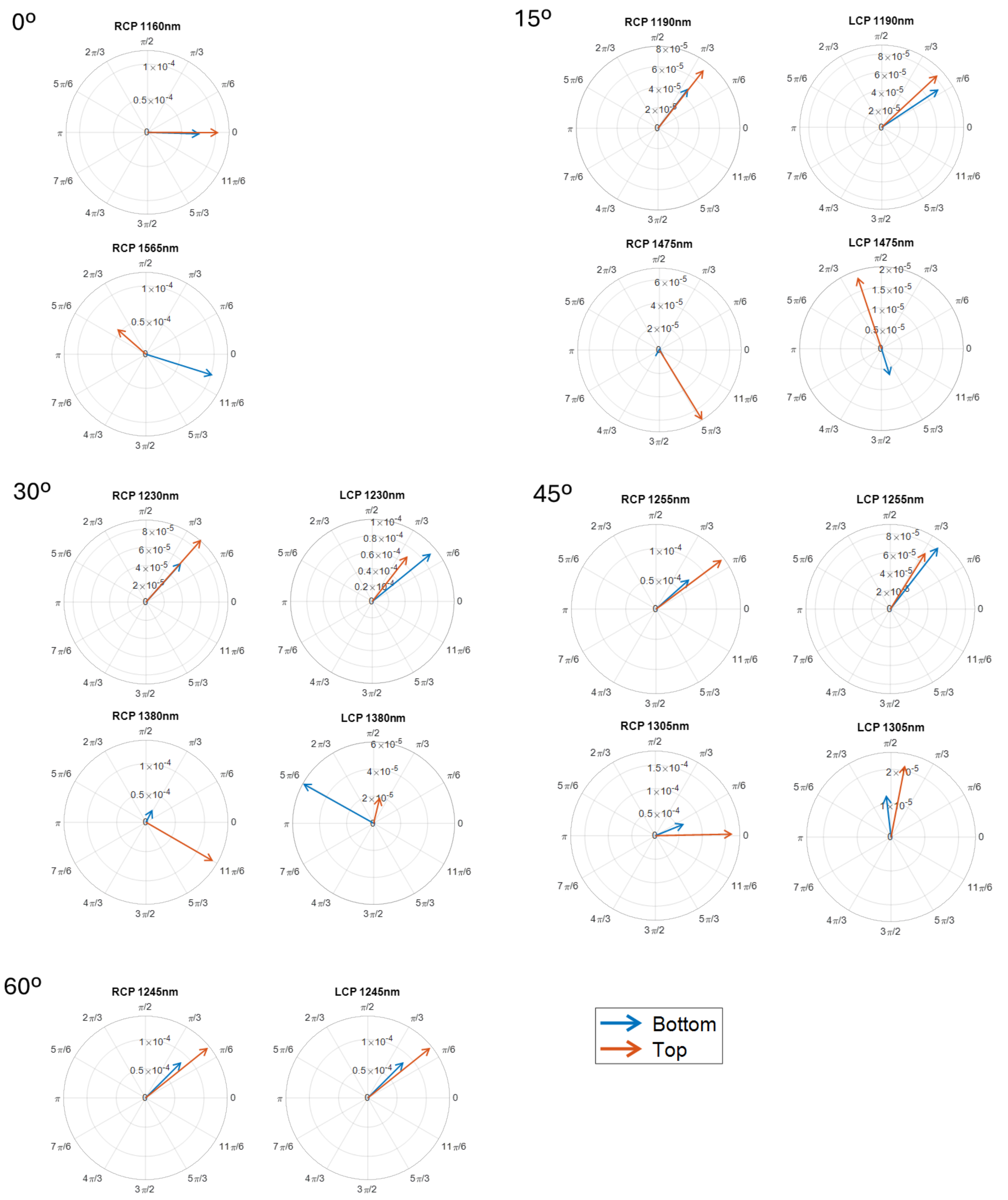

Fig. S6: Real part of the electric dipole moment computed for each of the two triskelia in the stack at an arbitrary phase of the incoming light. Data are extracted from FDTD simulations for twist angles of 0°, 15°, 30°, 45°, and 60° at wavelengths of interest under RCP and LCP. Note that for the 0° case, only RCP results are depicted since the stack has no definite handedness at this angle.

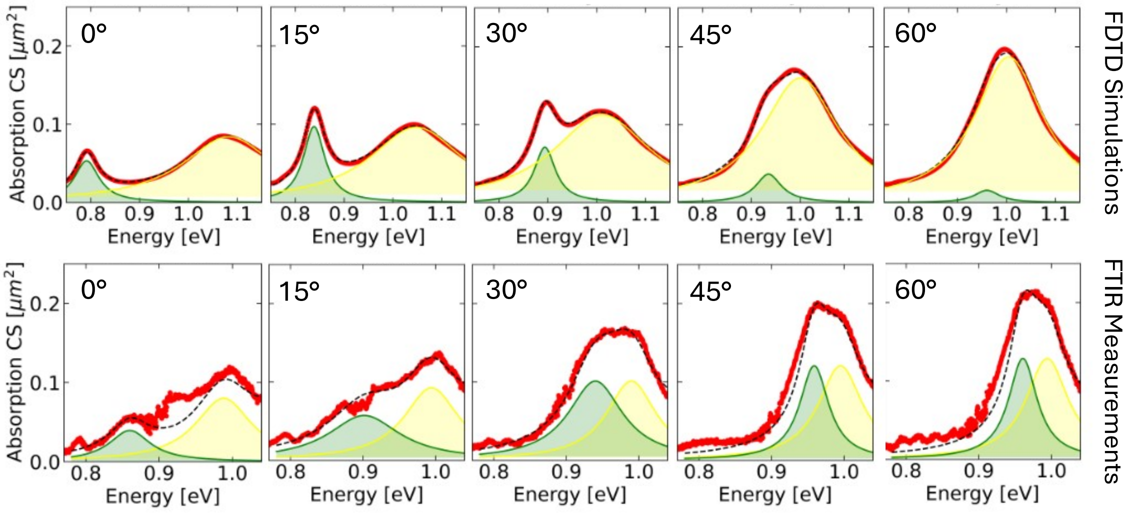

Fig. S7: Simulated (top row) and measured data (bottom row) fitted by the sum of two Lorentzian curves for several twist angles under RCP illumination.

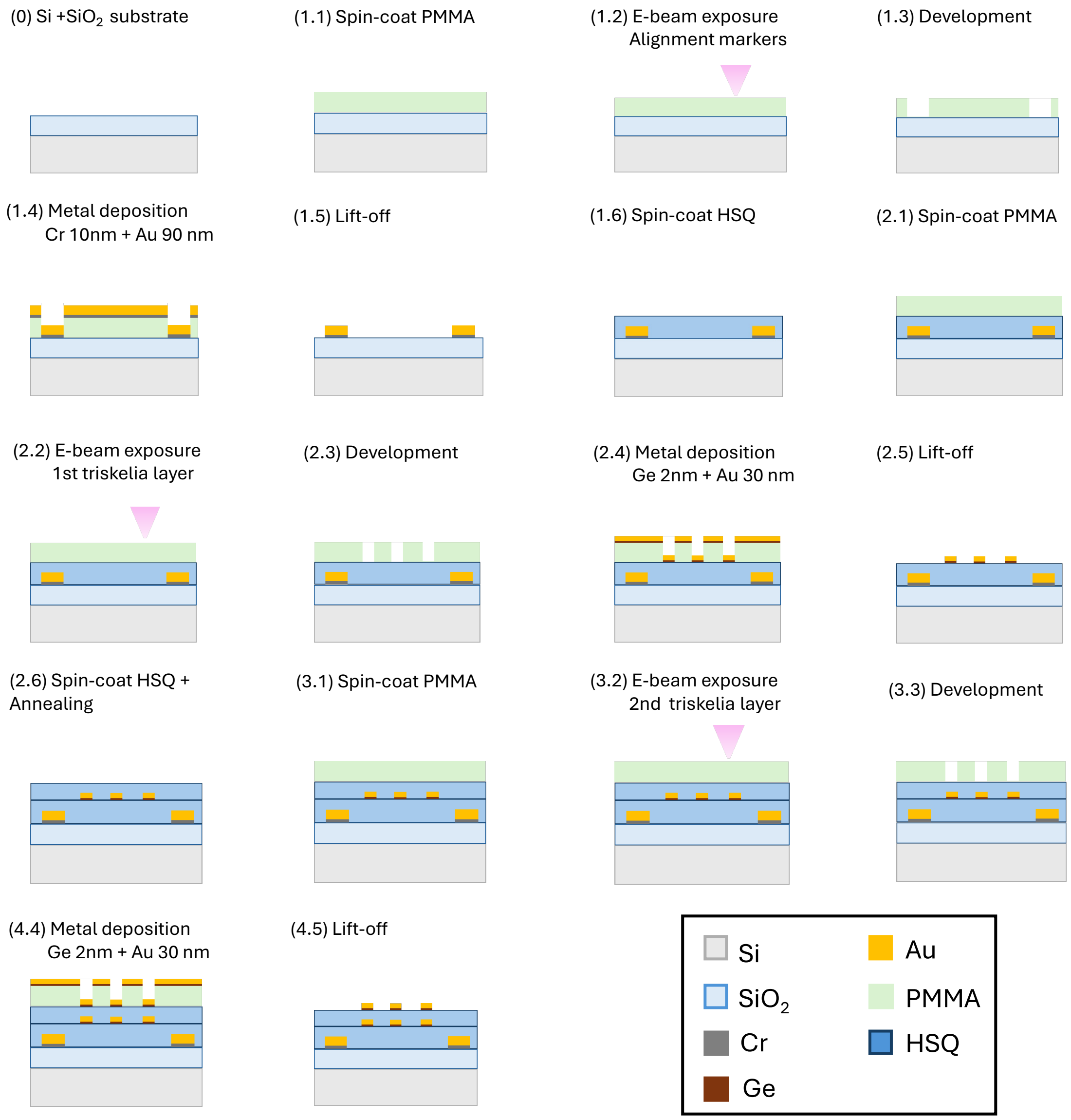

Fig. S8: Schematic representation of the necessary steps in the double lithography process used in the fabrication of the samples.

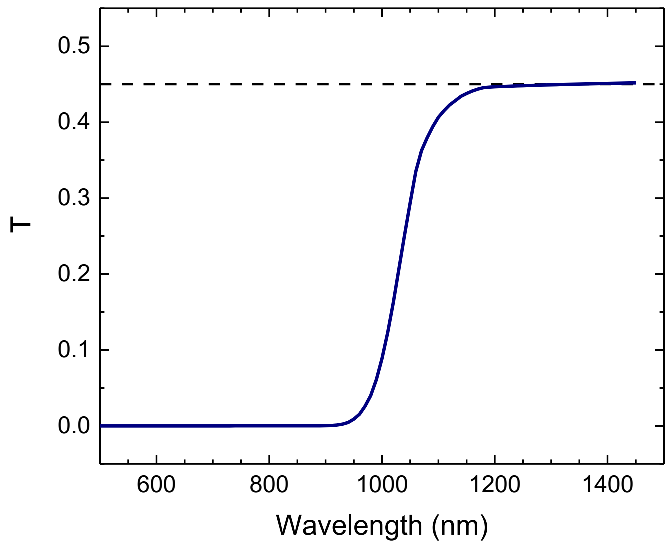

Fig. S9: Calculations of the transmission of the double oxidized Si substrate as a function of the wavelength. The transparency is almost 50% for wavelengths greater than 1200 nm. The calculation takes into account the energy lost by reflections in all the interfaces of the substrate as well as the absorption of the Si layer, using the optical constants for Si from the literature [1].

REFERENCES

[1] D. E. A. and A. A. Studna, "Dielectric functions and optical parameters of Si, Ge, GaP, GaAs, GaSb, InP, InAs, and InSb from 1.5 to 6.0 eV," *Phys. Rev. B*, vol. 27, no. 2, pp. 985–1009, 1983, doi: 10.1103/PhysRevB.27.985.
